# A comparative analysis of emotion recognition from EEG signals using temporal features and hyperparameter-tuned machine learning techniques

**DOI:** 10.1016/j.mex.2025.103468

**Published:** 2025-06-25

**Authors:** Rabita Hasan, Sheikh Md. Rabiul Islam

**Affiliations:** Department of Electronics and Communication Engineering (ECE), Khulna University of Engineering and Technology, Khulna 9203, Bangladesh

**Keywords:** Emotion classification, EEG signals, DEAP dataset, Machine learning, Feature extraction, Boosted EEG Emotion Classification Using Differential Entropy and Higuchi's Fractal Dimension

## Abstract

Classifying emotions based on EEG signals is really important for enhancing our interactions with computers, monitoring mental health and creating applications in affective computing field. This study explores improving emotion recognition performance by applying traditional machine learning classifiers and boosting techniques to EEG data from the DEAP dataset. To categorize emotional states, we used four classifiers: K-Nearest Neighbors (KNN), Support Vector Machine (SVM), XGBoost and Gradient Boosting. Differential entropy and Higuchi's fractal dimension are two important time-domain parameters that we extracted after applying a segmentation technique to capture the temporal interdependence of EEG data. These features were selected for their ability to reflect intricate neural dynamics associated with emotional processing. A five-fold cross-validation procedure was applied to estimate the model's performance and hyperparameter tuning was conducted to optimize classifier efficiency. XGBoost achieved the highest accuracy 89 % for valence and 88 % for arousal demonstrating its superior performance. Furthermore, cross-subject evaluation on the SEED dataset reinforced the approach’s robustness, where XGBoost achieved 86 % accuracy using HFD and 84 % using DE. These results emphasize the effectiveness of combining advanced feature extraction methods with boosting algorithms for EEG-based emotion recognition, offering promising directions for the development of real-world emotion-aware systems. The key findings of this research are as follows:

• Differential Entropy and Higuchi’s Fractal Dimension proved effective in capturing emotional brain dynamics
• XGBoost outperformed other classifiers in both DEAP and SEED datasets
• The proposed method demonstrates robustness across subject variations and datasets

## Graphical abstract

**Figure:**
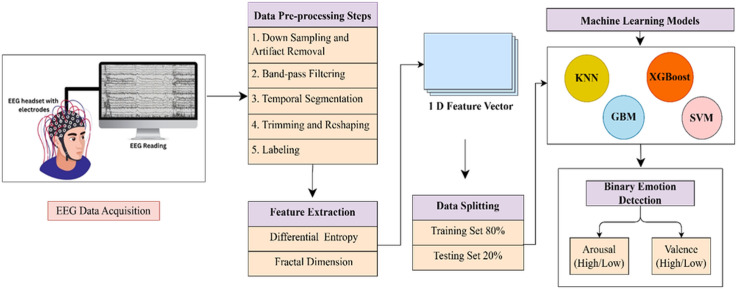

Specifications table

**Table:** 

Subject area:	Engineering
More specific subject area:	Signal Processing and Analysis, Features Extraction, Machine Learning Techniques
Name of your method:	Boosted EEG Emotion Classification Using Differential Entropy and Higuchi's Fractal Dimension
Name and reference of original method:	1. M. M. Rahman, A. K. Sarkar, M. A. Hossain, and M. A. Moni, "EEG-based emotion analysis using non-linear features and ensemble learning approaches," Expert Syst. Appl*.*, vol. 207, p. 118,028–118,040, 2022. https://doi.org/10.1016/j.eswa.2022.118025
Resource availability:	1. S.Koelstra et al., “DEAP: A database for emotion analysis; Using physiological signals,” IEEE Trans. Affect. Comput., vol. 3, no. 1, pp. 18–31, 2012. https://doi.org/10.1109/T-AFFC.2011.15 2. W.L. Zheng and B.L. Lu, ‘‘Investigating critical frequency bands and channels for EEG-based emotion recognition with deep neural networks,’’ IEEE Trans. Auton. Mental Develop., vol. 7, no. 3, pp. 162–175, Sep. 2015. https://doi.org/10.1109/TAMD.2015.2431497

## Background

Emotions are a vital part of how we connect with one another, significantly shaping our everyday experiences. An emotion detection system functions by using collected emotional response signals and features to assess human emotional states, as different emotions evoke different physiological and behavioral responses. Psychologists typically explain human emotions through two main models: the discrete emotion model and the dimensional model. The discrete model includes six basic emotions are happiness, sorrow, fear, anger, surprise and disgust, each associated with distinct facial expressions. In contrast, the dimensional model classifies emotions along continuous scales such as valence, arousal, and sometimes dominance.

One of the most widely adopted dimensional frameworks is Russell’s 2D emotion model, which considers only valence and arousal. Valence indicates the degree of pleasantness, ranging from negative to positive, while arousal represents the emotional intensity, ranging from low to high. In this study, we utilize the valence-arousal paradigm to describe and classify different emotional states.

Developing a reliable emotion detection system requires the ability to recognize, interpret and analyze various types of emotion-related data. Some systems rely on non-physiological cues such as speech [1], facial expressions [2] and body posture [3]. However, these signals are often influenced by cultural, age-related or individual behavioral differences, making it difficult to accurately interpret a person’s true emotional state. Therefore, physiological signals such as skin impedance [4], respiration and heart rate [5], magnetoencephalography (MEG) [6] and electroencephalography (EEG) [7] have been increasingly used in emotion detection systems. Physiological signals are more reliable due to direct links with emotional responses.

Among these, EEG is considered highly effective due to its portability, non-invasive nature, affordability and compatibility with wearable devices [[8], [9], [10]]. EEG captures the brain's electrical activity through electrodes positioned on the scalp. The resultant brainwave patterns, recorded and enhanced, yield substantial data for analyzing mental and emotional conditions [10].

In recent years, researchers have extensively explored machine learning algorithms and feature extraction techniques for EEG-based emotion detection, leveraging neural patterns to classify emotional states with high accuracy [11]. Notably, these models such as XGBoost, decision trees and stacking ensembles have also demonstrated remarkable performance in critical healthcare applications like stroke prediction, highlighting their versatility and potential in both neurological and clinical domains [12]. For example, [13] applied Hilbert-Huang Transform (HHT) to analyze EEG in the time-frequency domain, extracting instantaneous frequency features which were then classified using a multiclass SVM. Similarly, M. Menezes et al. [14] employed Russell’s Circumplex Model to guide their EEG-based affective analysis. They extracted features such as High Order Crossing and band power from α, β, δ, and θ waves using the DEAP dataset and classified them using SVM and Random Forest. In another approach, Veeramallu et al. [15] used the SEED dataset and Empirical Mode Decomposition (EMD) to classify emotions into positive, neutral and negative categories with a Random Forest classifier. In this study, we aim to improve EEG-based emotion recognition by integrating both traditional classifiers and boosting algorithms and our main contributions are:

I. Utilized KNN, SVM, XGBoost and Gradient Boosting on the DEAP dataset
II. Applied segmentation to extract Higuchi’s Fractal Dimension and Differential Entropy from EEG signals
III. Achieved 89 % accuracy using 5-fold cross-validation with XGBoost
IV. Reached 86 % and 84 % accuracy on SEED dataset using cross-subject validation

## Related work

An overview of current developments in emotion recognition is given in this section, which highlights the several approaches that have been employed to identify and categorize emotions utilizing EEG signals. Nevertheless, the majority of existing methods for identifying emotions from EEG signals depend on machine learning methods.

E. Hancer et al. [16] develops a robust EEG-based emotion recognition framework combining signal processing and ensemble learning. Leveraging advancements in portable and low-cost EEG devices, the framework includes preprocessing (multi-scale PCA and Symlets-4 filter), feature extraction (Dual Tree Complex Wavelet Transform), feature selection (statistical criteria) and classification using ensemble models. The random subspace ensemble classifier achieved a high accuracy of 96.8 %, indicating the framework's strong performance in recognizing emotions. This comprehensive pipeline demonstrates the potential of integrating advanced signal processing and machine learning techniques to enhance the accuracy and reliability of EEG-based emotion detection. Another method that successfully combines the crucial phases of EEG signal processing with the extraction of important features was created by Alhalaseh et al. [17]. In this work, empirical mode decomposition and variational mode decomposition was applied for signal analysis purposes. Higuchi's Fractal Dimension and entropy approaches were used in the feature extraction procedure. The DEAP database was employed to categorize emotions using KNN, naive Bayes, decision tree and convolutional neural network (CNN) classifiers. Applying the CNN classifier, researchers attained a precision of 95.20 %.

Using specific EEG channels and frequency bands, Z. Mohammadi et al. [18] examined emotional states within the arousal-valence dimensions. The researchers in this study extracted a number of features after utilizing the DWT technique to separate the relevant frequency bands from the EEG data. SVM and KNN models are employed to discern emotional statuses. The 10-channel EEG data achieved an impressive accuracy for both arousal and valence state, they are 86.75 % and 84.05 %, respectively. Statistical time-domain characteristics were retrieved for emotion recognition in a model created by R. Nawaz et al. [19]. Principal Component Analysis (PCA) was utilized to improve performance accuracy. The SVM was employed to assess the efficacy of the features, with a peak classification accuracy of 78.06 %.

Y. Fang et al. [20] introduced a multi-feature deep forest (MFDF) model for human emotion recognition. The EEG data are first split into numerous frequency sub-bands, from which parameters like PSD and differential entropy are obtained. Then five different emotions are categorized within a five-class emotion framework. To classify these emotions, a deep forest model is constructed utilizing the extracted features. The experiments exploited a publicly accessible dataset for physiological signal-based emotion analysis (DEAP). Traditional classifiers, including SVM, Random Forest and KNN are employed to evaluate the results. The MFDF strategy attained an average classification accuracy of 71.05 %.

Asymmetric EEG patterns from several experimental sessions were used by Zheng et al. [21] to create a emotion detection model. This research utilized various feature selection techniques and binary classification to detect emotions. The original maximum accuracy achieved using a SVM classifier with RASM features was 51.83 %. With the use of DE features, the incorporation of graph-regularized extreme learning machine (GELM) generated an improved accuracy of 69.67 %. This research emphasized the significance of asymmetric patterns and sophisticated feature collection systems for emotion recognition. To address the issue of insufficient data in emotion detection tasks, a synthesis of three well-known datasets DREAMER, DEAP and a proprietary dataset gathered by the researchers is offered in [22]. This consolidated dataset comprises 60 people, the highest count among current datasets. The approach attains accuracies of 70.26 % for valence and 72.42 % for arousal. The research in [23] advocates for an EEG-based emotion detection technique employing EMD and Approximation Entropy to derive features from the initial four Intrinsic Mode Functions (IMFs). A Deep Belief Network integrated with a SVM is exploited to classify four emotions: happiness, calmness, sadness and fear. Experiments employing 16-lead EEG data attained an average accuracy of 83.34 % and a peak accuracy of 87.32 %, indicating competitive efficacy relative to leading methodologies.

To identify human emotions, some researchers have conducted tests with hybrid traits. A. M. Bhatti et al. [24] established a methodology that incorporates hybrid features from three different domain applying audio music as the external stimulus. This work exploited a multi-layer perceptron (MLP) classifier to recognize seven unique emotions, attaining an accuracy of 94 %. Md. Khateeb et al. [25] developed a feature fusion model for emotion classification with the DEAP dataset, focusing on four channels: FP1, FP2, F3 and C4. This study examined multi-domain characteristics from the temporal, wavelet and frequency domains. The collected characteristics were further trained using an SVM classifier to classify nine distinct emotion categories, attaining an accuracy of 65.92 %.

The literature review presented above is summarized in Table 1, which also highlights the goals, approaches, findings and unmet research needs of recent studies on EEG-based emotion identification.

**Table 1:** Summary of review papers on EEG-based emotion recognition.

Ref	Objective	Methodology	Outcome	Research gap
[16]	Develop EEG-based emotion recognition framework leveraging signal processing and ensemble learning.	Multi-scale PCA and Symlets-4 for preprocessing, DTCWT for feature extraction, statistical feature selection, and ensemble classifiers for classification.	Achieved accuracy of 96.8 % using the Random subspace ensemble classifier.	Lacks of cross-dataset validation and real-time implementation analysis.
[18]	Investigate emotional states within arousal-valence dimensions using selected EEG channels.	DWT was used for frequency band decomposition, machine learning models (SVM and KNN) were utilized for emotion classification.	86.75 % of arousal accuracy and 84.05 % of valence with 10-channel EEG data.	Limited to selected EEG channels, needs exploration on a full-channel dataset for better performance.
[19]	Develop a model for emotion recognition using statistical time-domain features	Statistical features were extracted and PCA was applied for dimensionality reduction, SVM was used for classification.	Maximum accuracy of 78.06 % was achieved.	Further work on advanced feature extraction and deep learning methods is suggested.
[20]	Propose a multi-feature deep forest model for detecting human emotions.	Features like DE and PSD were extracted from frequency bands, a deep forest model was developed and compared with conventional classifiers like SVM, RF and KNN.	Achieved 71.05 % average recognition accuracy.	Investigating alternative feature sets and hybrid deep learning models could enhance performance.
[21]	Develop an EEG-based emotion recognition system leveraging asymmetric EEG patterns.	Different feature selection methods were used to extract asymmetric patterns; a SVM classifier was initially applied, followed by graph-regularized extreme learning machine (GELM).	SVM achieved 51.83 % accuracy with RASM features, GELM improved accuracy to 69.67 % using DE features.	Limited to specific feature selection methods, further exploration with advanced techniques and larger datasets is required.
[22]	Solve the problem of inadequate data in emotion detection tasks by combining multiple datasets.	Combined data from DEAP, DREAMER and a private dataset, utilized SVM to analyze time-frequency EEG features.	Achieved an accuracy of 70.26 % for valence and 72.42 % for arousal across 60 participants.	The accuracy results should be further improved.
[23]	Develop an EEG-based emotion recognition method leveraging EMD and ApEn for feature extraction.	EMD was applied to decompose EEG signals, ApEn was computed for the first four IMFs as features. A Deep Belief Network combined with SVM was used for classification.	Achieved an average accuracy of 83.34 % and a maximum of 87.32 %.	Further research is needed to evaluate performance on larger datasets and explore alternative classifiers.
[24]	Detect emotions using hybrid features from different domains.	An external stimulus (audio music) was used as an external stimulus, hybrid features were extracted and classified using an MLP classifier.	Achieved 94 % accuracy for identifying seven distinct emotions.	Limited to audio music stimuli, lacks analysis of diverse emotional stimuli.
[25]	Develop a multi-domain feature fusion model for emotion classification.	Features from time, wavelet and frequency domains were fused, four EEG channels were selected and an SVM classifier was applied.	Achieved 65.92 % accuracy for classifying nine emotion classes using the DEAP dataset.	Restricted to four EEG channels, extending the study to all channels and more advanced classifiers is needed.

Unlike previous studies that compute global features across entire EEG trials without accounting for temporal dependencies, our framework adopts a segmentation-based approach to capture localized temporal variations in brain activity. By extracting dynamic complexity through Higuchi’s Fractal Dimension and uncertainty via Differential Entropy, we enrich the feature space with both nonlinear and temporal descriptors. Additionally, we conducted correlation and distribution analysis of DE and HFD features across segments, uncovering distinct structural patterns that enhance their discriminative power in emotion classification. Furthermore, we integrate boosting algorithms with conventional classifiers to enhance the robustness of emotion detection, while effectively addressing the overlooked temporal dynamics crucial for accurate EEG-based emotion recognition.

## Method details

**Fig. 1:**
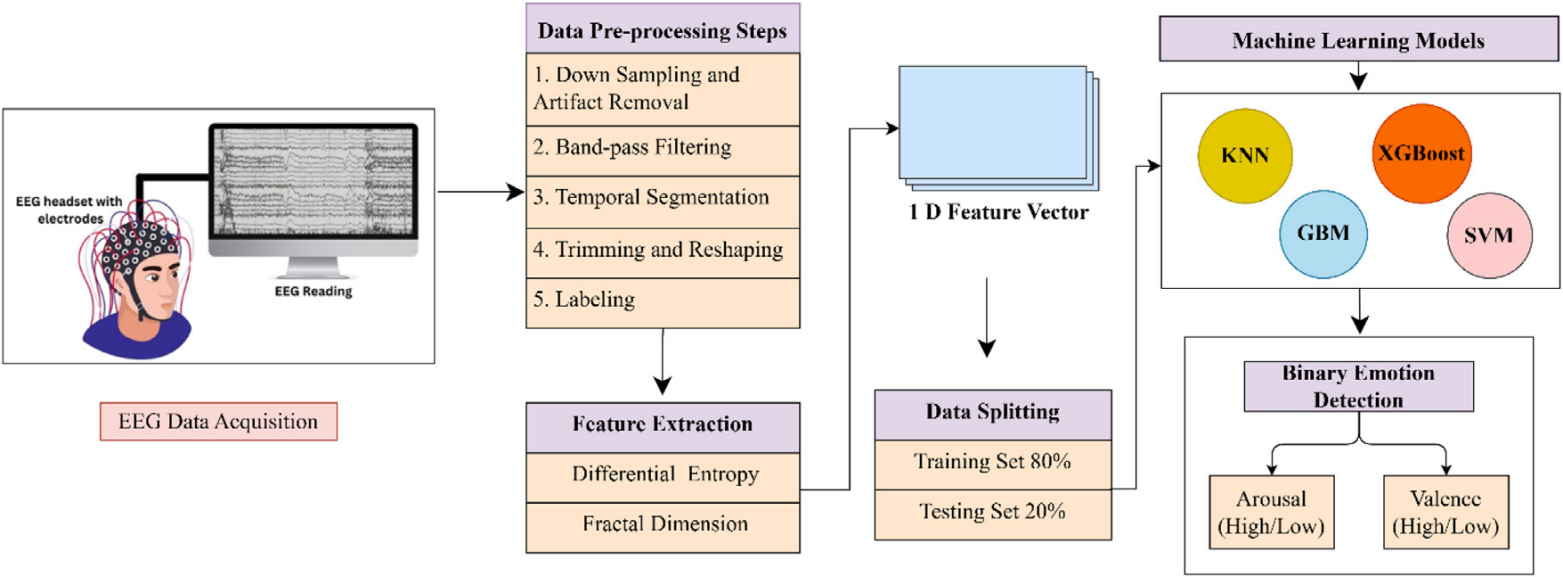
The consecutive steps of our proposed model involve reshaping, segmentation, feature extraction and classification to identify emotions from raw EEG signals.

The proposed model's block diagram, which classifies emotional states as an output and employs EEG data as an input parameter, was displayed in Fig. 1. After that, each part of the model is explained in detail.

### Dataset explanation

The DEAP dataset is an easily accessible resource for emotion analysis based on physiological signals [26]. It includes EEG, physiological and frontal facial video data from 32 participants and aged between 19 – 37 years old. EEG recordings were conducted utilizing the 10/20 international arrangement with 32 channels sampled at 512 Hz, along with 12 peripheral channels, 1 status channel and 3 unused channels (total: 48 channels). Participants' physiological reactions and self-reported emotional ratings were recorded as they saw 40 one-minute music videos. A thumbs-up or thumbs-down approach was used to measure liking, and the Self-Assessment Manikin (SAM) was used to rate emotions on a scale of 1 to 9 for valence, arousal and dominance. Familiarity was assessed on a 1–5 scale. The DEAP dataset array is structured as video × channel × data = 40 × 40 × 8064 and labels shape as video/trial × label (valence, arousal, dominance, liking) = 40 × 4.

### Data cleaning

In our experiment, the EEG data was retrieved from DEAP’s official website and this dataset is available in both raw and pre-processed format. To improve the quality of raw data and prepare it for further analysis, data preprocessing steps are essential. This procedure ensures the extraction of significant insights, increases model performance and improves data reliability. In our study, five vital preprocessing steps are applied including down sampling and artifact removal, filtering technique, temporal segmentation, reshaping and trimming, labeling.

### Down sampling and artifact removal

**Fig. 2:**
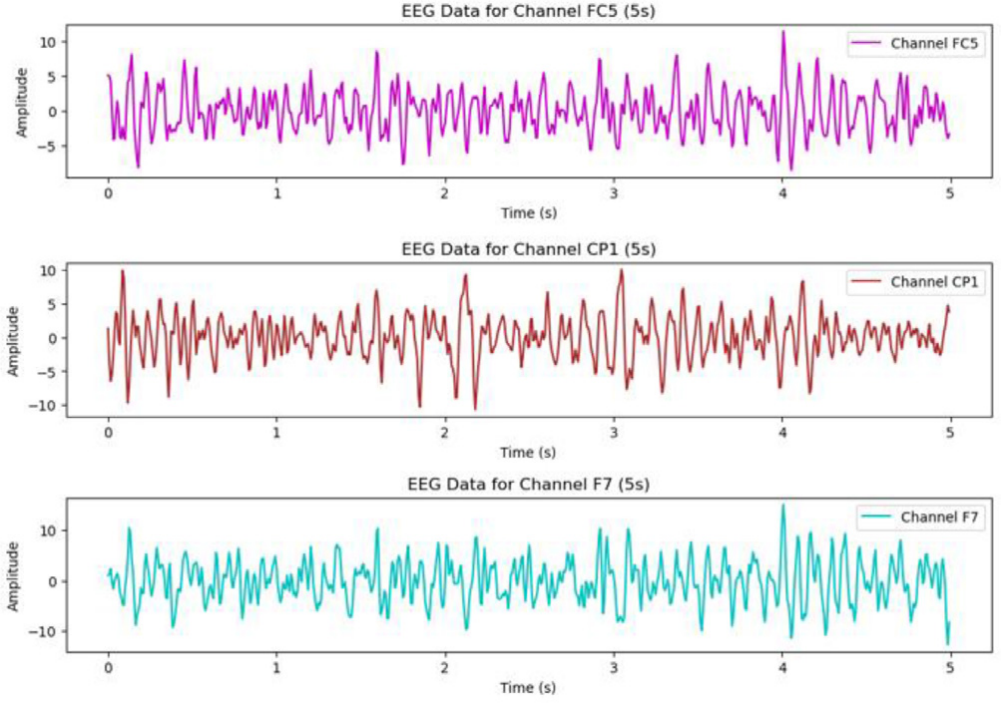
DEAP EEG data after pre-processing.

The raw EEG data initially recorded at 512 Hz, was down-sampled to 128 Hz to reduce data volume and processing load while preserving the essential characteristics of the signals. This down-sampling technique also played a crucial role in preventing aliasing, which helped maintain the quality of the EEG recordings. As a result, it made the analysis more efficient and aided in eliminating artifacts. Fig. 2 illustrated raw EEG data from different channels after filtering and eliminating artifacts.

### Bandpass filtering

**Fig. 3:**
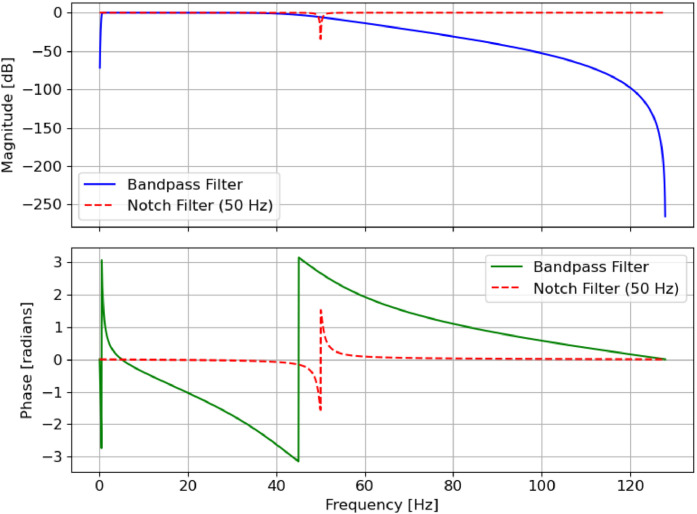
The frequency and phase response of a 4th-order Butterworth bandpass filter and a 50 Hz notch filter.

A 4th-order Butterworth band-pass filter with a frequency range of 4 Hz to 45 Hz was then applied to the EEG data. Both noise and EOC artifacts are lessened as a result. In order to eliminate high-frequency noise and low-frequency drifts, the bandpass filter is made to keep EEG components between 0.5 Hz and 45 Hz. To get rid of powerline interference, a notch filter is used at 50 Hz. Fig. 3 presents the frequency response of a bandpass and notch filter used for EEG signal preprocessing.

The top plot displays the magnitude response, showing that the bandpass filter attenuates frequencies outside the 0.5–45 Hz range, while the notch filter effectively suppresses the 50 Hz powerline noise. The bottom plot illustrates the phase response, demonstrating how these filters affect the phase characteristics of the EEG signal. In addition, to improving the signal quality, this filtering procedure yields more pertinent EEG data for emotion identification.

### Segmentation technique

**Fig. 4:**
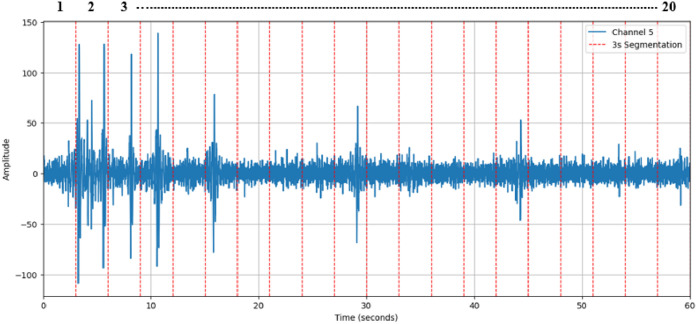
A 60 s. EEG signal is divided into 20 segments.

Segmentation is a fundamental step in EEG signal analysis for emotion detection because it helps analyzing temporal dynamics and also extracting meaningful features. Emotions can induce fluctuating changes in brain activity over time. By dividing the EEG signal into segments researchers can examine these time-dependent variations and revealing how emotional responses evolve, develop and subside within different periods. In DEAP dataset, a single EEG signal’s length is 60 s. This 60 s. EEG signal is divided into 20 portions and each segment contains data of 3 s. So total segmented number will be 25,600. A single EEG channel segmentation process is demonstrated in Fig. 4.

### Trimming and reshaping

The downloaded DEAP dataset was structured in the following format: video × channel × data = 40 × 40 × 8064. This indicated that there were 40 distinct videos, 40 channels and 8064 (128 Hz × 63 s.) data points. Among these, the initial 32 channels continuously recording the raw EEG signals from human brain and significant features were extracted from these 32 channels exclusively. Additionally, after removing the first 3 s of pre-trial data the remaining data points is 7680 (128 Hz × 60 s.), which is known as trimming method. Then the reshaped data to a format of video × channel × data = 40 × 32 × 7680.

### Data labeling

In the last step of preprocessing, we labeled the data according to how participants evaluated their emotions in terms of valence and arousal. These ratings were ranging from 1 to 9-point scale, which indicated the strength of their emotional reactions. To make classification easier, we used a binary labeling system:

• Ratings of 4.5 or lower were marked as 0 (signifying a low emotional response)
• Ratings above 4.5 were marked as 1 (indicating a high emotional response)

We chose this threshold based on previous research in EEG-based emotion recognition, which often employs similar binary classifications. This binary method not only simplifies the learning process but also makes the model easier to understand and boosts the efficiency of training and evaluation in machine learning systems aimed at recognizing emotions.

### Feature extraction engineering

**Fig. 5:**
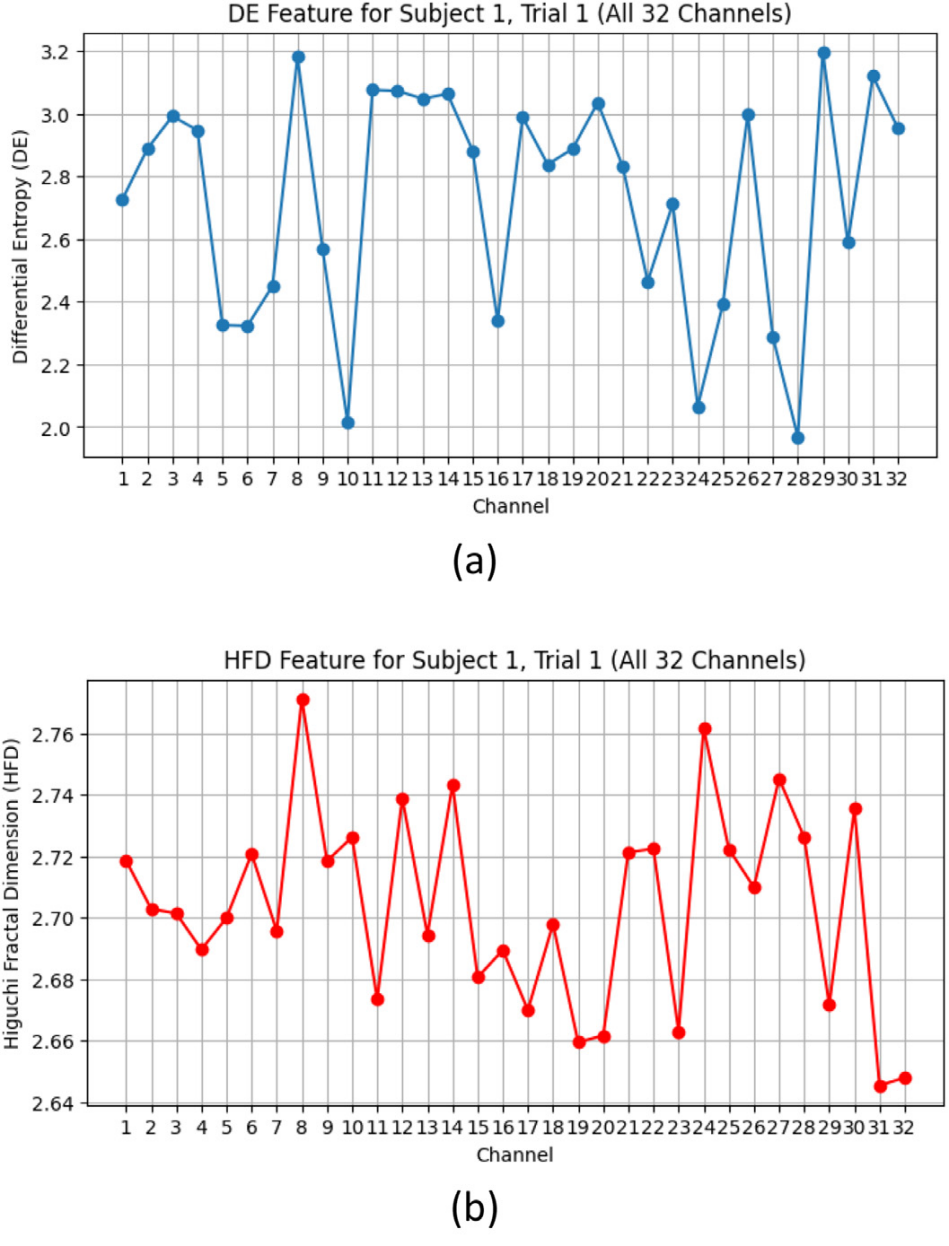
Graphical Representation of Extracted Features: (a) Differential Entropy (DE) Feature (b) Higuchi's Fractal Dimension (HFD) Feature, for Subject 1, Trial 1, all 32 Channels.

The process of turning raw data into a collection of valuable properties or features is known as feature extraction and it is an essential step in machine learning and data analysis. In order to properly record and characterize the behavior of brain electrical activity across a range of emotional states, specific features must be extracted in order to construct an emotion identification system. We used Higuchi's approach to estimate the fractal dimension and a histogram-based probability distribution function to extract the differential entropy feature for emotion recognition. Fig. 5 illustrates the graphical representation of DE and HFD features for Subject 1, Trial 1, all 32 Channels of DEAP dataset.

### Histogram-based probability distribution to calculate differential entropy

According to earlier research, DE is the most reliable and accurate EEG feature for detecting emotions [20,27]. Differential entropy quantifies the uncertainty of a continuous random variable, reflecting the extent of unpredictability in its potential outcomes. The histogram-based probability distribution method assesses the signal's probability distribution by calculating a normalized histogram for each 3-second segment of EEG data. The 'numpy. histogram ()' function in Python 3 is used to compute the histogram. To guarantee an adequate number of bins for accurately representing the distribution without overfitting or underfitting, the parameter ‘bins = auto’ autonomously determines the appropriate number of bins based on the data distribution. The histogram, normalized with the 'density=True' parameter, shows the probability density function (PDF), essential for entropy calculation. The mathematical formula of differential equations is defined as:

(1) $$h\left(X\right)=-\sum_{i=1}^{M}p\left(x_{i}\right)logp\left(x_{i}\right)\;\Delta \;x\;$$

where, X signifies random variable, $p(x_{i})$ represents probability density of each bin and Δx is the bin width. The summation approximates the continuous integral for differential entropy. A time series X that follows a Gaussian distribution N (µ, σ²), then the differential entropy can be written in the following format:

(2) $$\begin{array}{c} h\left(X\right)=-\int_{-\infty }^{\infty }\frac{1}{\sqrt{2\pi \sigma^{2}}}e^{-\left[\frac{{\left(x-\mu \right)}^{2}}{2\sigma^{2}}\right]}\log\left[\frac{1}{\sqrt{2\pi \sigma^{2}}}e^{-\left[\frac{{\left(x-\mu \right)}^{2}}{2\sigma^{2}}\right]}\right]dx\\ \;\;\;\;\;\;\;\;\;\;\;\;=\frac{1}{2}\log\left(2\pi e\sigma^{2}\right) \end{array}$$

### Higuchi's algorithm to calculate fractal dimension

The fractal dimension of an EEG signal quantifies the signal's complexity and self-similarity across various temporal scales. It evaluates the complexity or irregularity of the EEG waveform, reflecting elements of its temporal structure that conventional statistical techniques do not reveal. Prior studies have indicated that FD characteristics has the capability for emotion identification exploiting EEG signals [[28], [29], [30]]. In this study, we employed a method devised by Higuchi to directly calculate the fractal dimension (FD) and the mathematical expression:

(3) $$X_{i}^{j}=X\left(i\right),X\left(i+j\right),X\left(i+2j\right),\ldots \ldots .X\left(i+\text{int}\left(\frac{N-i}{j}\right)\times j\right)$$

where N is the total number of samples, i is the beginning time, j is the internal time, and *i* = 1, 2, 3, 4…..j.

Now, the curve length equation is:

(4) $$L_{i}\left(j\right)\;=\;\frac{\sum_{m=1}^{\text{int}\left(\frac{N-i}{j}\right)}\left|X\;\left(i+\text{mj}\right)-\;X\left(i+m-1\right)j\right|\times \left(n-1\right)}{k\;\times \;\text{int}\;\left(\frac{N-i}{j}\right)}\;$$

The HFD method is calculated using the following equation:

(5) $$\text{HFD}\;=\;\frac{L\left(j\right)}{\text{log}\;j}\;$$

### Feature correlation and distribution analysis

**Fig. 6:**
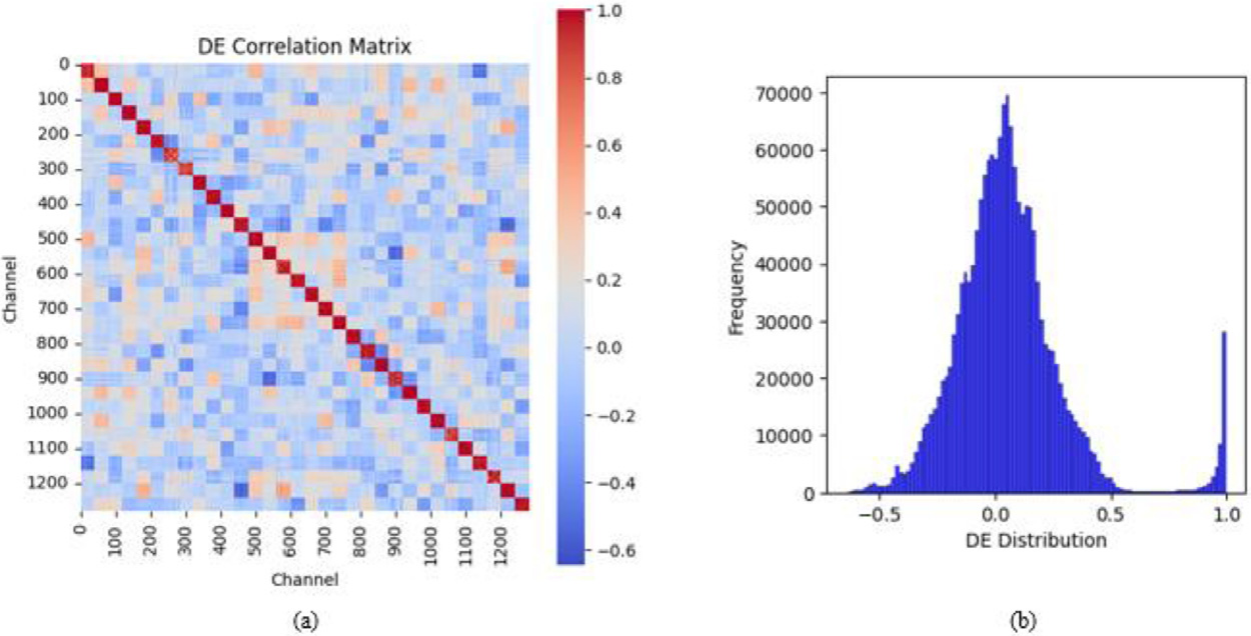
(a) Correlation matrix of DE feature (b) Distribution plot of DE feature.

**Fig. 7:**
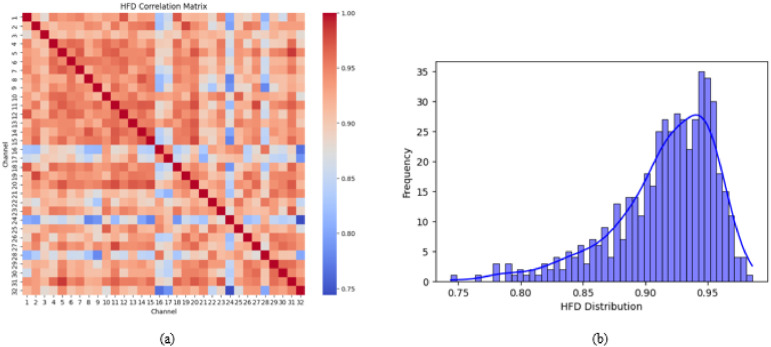
(a) Correlation matrix of HFD feature (b) Distribution plot of HFD feature.

The correlation matrices for DE and HFD, Fig. 6(a) and Fig. 7(a) illustrate the relationship between feature values across different channels. The DE correlation matrix reveals a mix of positive and negative correlations, with a diagonal dominance indicating self-correlation. Conversely, the HFD correlation matrix shows predominantly high positive correlations across channels, suggesting a more homogeneous feature representation.

The distribution plots, Fig. 6(b) and Fig. 7(b) depict the statistical distribution of DE and HFD values. The DE feature distribution follows a near-Gaussian shape, with a peak around zero and a heavy-tailed structure. In contrast, the HFD feature distribution exhibits a right-skewed pattern, with values clustering between 0.75 and 0.95. A fitted density curve further highlights the underlying statistical trends in both distributions. These analyses provide insights into the structural properties of the extracted features, offering a foundation for their role in classification tasks.

### Machine learning models

To independently forecast the emotion accuracy results, we used four distinct machine learning models in our study: KNN, SVM, XGBoost and Gradient Boosting. The comprehensive mathematical procedures of four distinct machine learning models related to the emotion detection process are represented in Algorithm 1.

**Algorithm 1:** Mathematical representation of machine learning models.

## Method validation

The performance of the suggested models is thoroughly compared in this section. We start by describing the evaluation measures that are used to determine how effective the procedures that have been put into practice are. After that, we give a thorough explanation of the experimental design and a detailed analysis of the outcomes. Lastly, we highlight the advantages and competitiveness of our methodology by contrasting our classification results with a number of cutting-edge techniques.

### Evaluation metrics

The performance of the proposed model in emotion detection tasks is evaluated using several standard metrics and the formula for calculating each matrix is detailed below. Accuracy is a crucial evaluation parameter that reflects the overall performance of a model. The definition is the ratio of correctly anticipated cases to the total instances. The subsequent equation presents a formal declaration of the accuracy equation.

(6) $$Accuracy\;=\frac{TP+TN}{TP+TN+FP+FN}\;$$

Another significant metric is precision, defined as the ratio of correctly predicted positive observations to the total number of expected positives. The precision underscores the accuracy of favorable projections. The following mathematical statement represents precision:

(7) $$Precision\;=\frac{TP}{TP+FP}\;$$

The ratio of accurately predicted positive instances to the total number of actual positive instances is known as recall or sensitivity. It underscores the model's capacity to encompass all positive instances. Equation 08 articulates the recall formula as follows.

(8) $$Recall\;=\frac{TP}{TP+FN}$$

The harmonic mean of recall and precision is represented by the F1 score. When there is an unequal distribution of classes, this measure is very helpful because it provides a fair assessment. Equation 09 represents the mathematical formulation of the F1 score.

(9) $$F1\;Score\;=\frac{2\;\times Precision\times Recall}{Precision+Recall}\;$$

where the symbols TP, TN, FP and FN signifies the corresponding numbers of true-positives, true-negatives, false-positives and false-negatives.

### Experimental setup

**Fig. 8:**
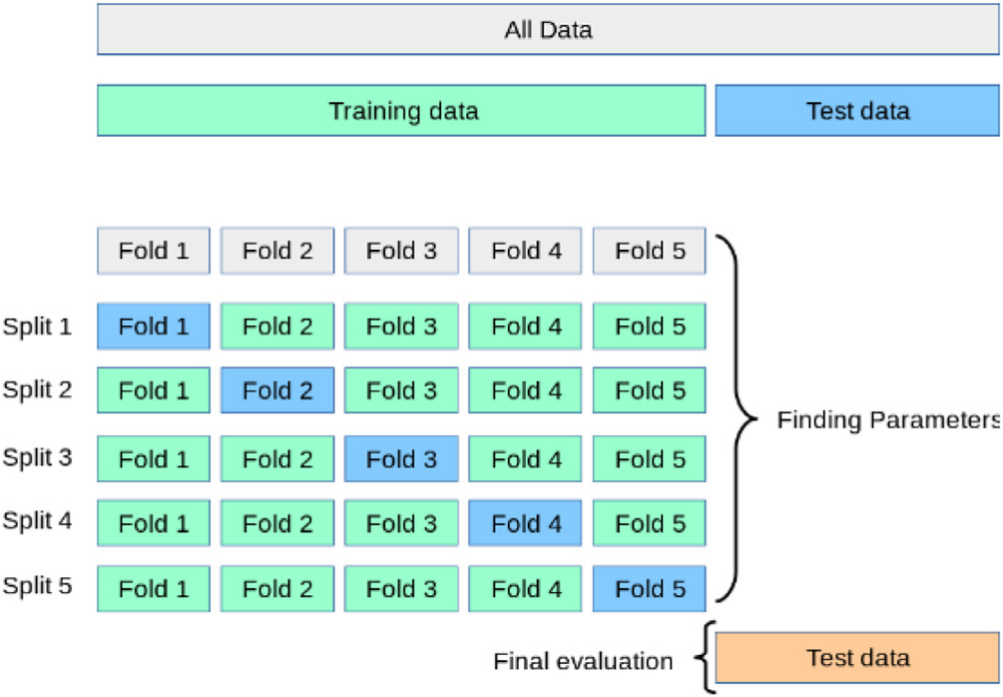
A partitioning technique for 5-fold cross validation [32].

We conducted our experiment employing 'DEAP' dataset of EEG motions to identify and classify emotions. After splitting the EEG data into 20 temporal segments, each applicant viewed 40 distinct emotional movies, for a total of 800 segments (40 × 20). For 32 participants, 800 × 32 = 25,600 segments were generated. This EEG data is then used to extract two significant features, DE and HFD. In this experiment, we identify valence and arousal state from the DEAP dataset using the binary classification technique. We utilize the number "4.5″ as a threshold to distinguish between the valence and arousal level binary categorization. The following scale is used to identify the levels as: low value ranges from 0 to 4.5 and high value ranges from 4.5 to 9. In the binary classification investigation, 80 % of the data was used for training and 20 % for testing in a 5-fold cross-validation process. A statistical technique called cross-validation divides the data into subgroups in order to evaluate the effectiveness of a machine learning model. In this technique, the dataset is divided into five subsets with four used for model training and the fifth for testing in each iteration. This technique facilitated optimal use of the available data, allowing for a complete evaluation of the model's performance in all dimensions [31]. Fig. 8 illustrates the 5-fold cross-validation partitioning system.

### Experimental result

This study aims to develop a segmentation-based framework that captures temporal variations in EEG signals, facilitating the extraction of localized dynamic and nonlinear features. The framework is further strengthened by correlation and distribution analysis to uncover structural patterns that improve emotion classification. Additionally, boosting algorithms are integrated with conventional classifiers to enhance overall model performance. A complete explanation of the particular results attained is provided below. Table 2, Table 3 represents the overall classification reports for differential entropy and fractal dimension respectively.

**Table 2:** Classification outcomes of differential entropy feature.

ML Model	Valence / Arousal	Accuracy	Class 0 = Low, 1 = High	Precision	Recall	F1 Score	Support
KNN	Valence	0.72	0	0.71	0.59	0.64	2178
			1	0.73	0.82	0.77	2942
	Arousal	0.70	0	0.67	0.66	0.67	2296
			1	0.73	0.74	0.73	2824
SVM	Valence	0.63	0	0.71	0.23	0.35	2178
			1	0.62	0.93	0.74	2942
	Arousal	0.60	0	0.71	0.18	0.29	2296
			1	0.58	0.94	0.72	2824
XGBoost	Valence	**0.89**	0	0.94	0.79	0.86	2178
			1	0.86	0.96	0.91	2942
	Arousal	**0.88**	0	0.93	0.81	0.86	2296
			1	0.86	0.95	0.90	2824
Gradient Boosting	Valence	0.63	0	0.77	0.17	0.28	2178
			1	0.61	0.96	0.75	2942
	Arousal	0.60	0	0.70	0.18	0.29	2296
			1	0.58	0.94	0.72	2824

**Table 3:** Classification outcomes of Higuchi's fractal dimension feature.

ML Model	Valence / Arousal	Accuracy	Class 0 = Low, 1 = High	Precision	Recall	F1 Score	Support
KNN	Valence	0.78	0	0.76	0.71	0.74	2178
			1	0.80	0.84	0.82	2942
	Arousal	0.77	0	0.75	0.71	0.73	2296
			1	0.78	0.81	0.79	2824
SVM	Valence	0.57	0	0	0	0	2178
			1	0.57	1	0.73	2942
	Arousal	0.59	0	0.60	0.27	0.37	2296
			1	0.59	0.85	0.70	2824
XGBoost	Valence	**0.86**	0	0.90	0.76	0.82	2178
			1	0.84	0.94	0.89	2942
	Arousal	**0.86**	0	0.89	0.80	0.84	2296
			1	0.85	0.92	0.88	2824
Gradient Boosting	Valence	0.66	0	0.72	0.34	0.46	2178
			1	0.65	0.90	0.75	2942
	Arousal	0.64	0	0.68	0.36	0.47	2296
			1	0.62	0.86	0.72	2824

From Table 2, we observed that the differential entropy feature achieved 89 % accuracy in valence state classification and 88 % accuracy in arousal state classification using the XGBoost algorithm. The high F1 score indicates a satisfactory balance between precision and recall. In contrast when using this feature, both the SVM and gradient boosting models achieved lower accuracies of 63 % and 60 % for valence and arousal states, respectively. In Table 3, we examined the Higuchi's fractal dimension feature which achieved 86 % accuracy for both valence and arousal states with the XGBoost algorithm. However, the SVM performed worse with this feature attaining accuracies of 57 % and 59 % for valence and arousal states, respectively. Overall, it's evident that for both the differential entropy and Higuchi's fractal dimension features the XGBoost algorithm yielded the highest accuracy, with differential entropy being the optimal feature.

Differential entropy is considered the optimal feature due to its high discriminative power which effectively captures the complexity and variability of the underlying data. This characteristic allows it to differentiate between various emotional states such as valence and arousal with greater accuracy. Additionally, differential entropy is robust to noise and outliers leading to more reliable classification results. It quantifies the amount of uncertainty in a signal providing rich information that correlates well with emotional states. This feature also aligns well with the XGBoost algorithm enhancing its performance through the nature of the data it represents. Moreover, the high F1 score associated with differential entropy indicates a satisfactory balance between precision and recall making it an effective choice for classification tasks. Overall, these factors contribute to differential entropy’s status as the optimal feature in emotion recognition.

**Fig. 9:**
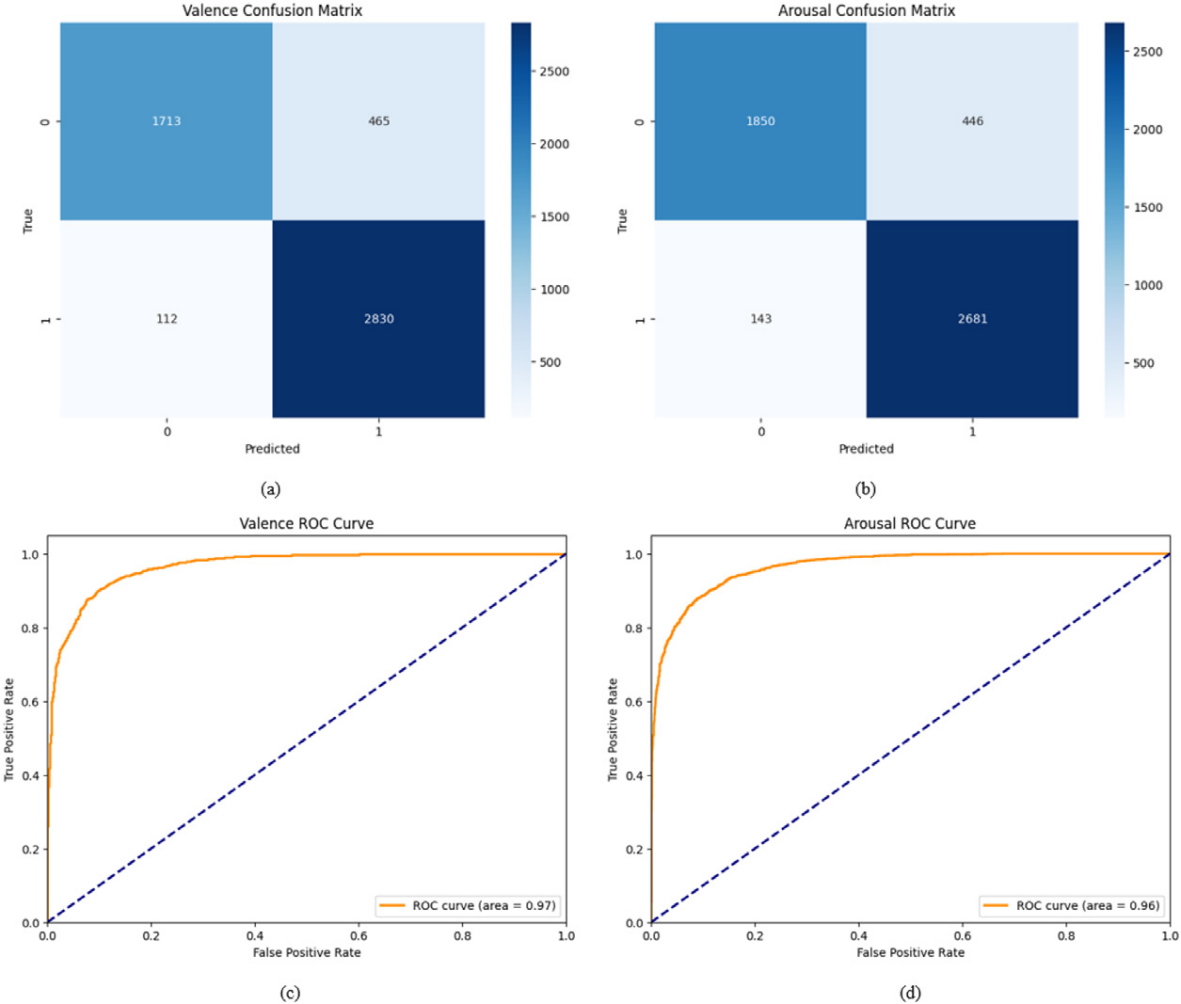
(a-b) Confusion matric and (c-d) ROC curve of differential entropy using XGBoost algorithm.

**Fig. 10:**
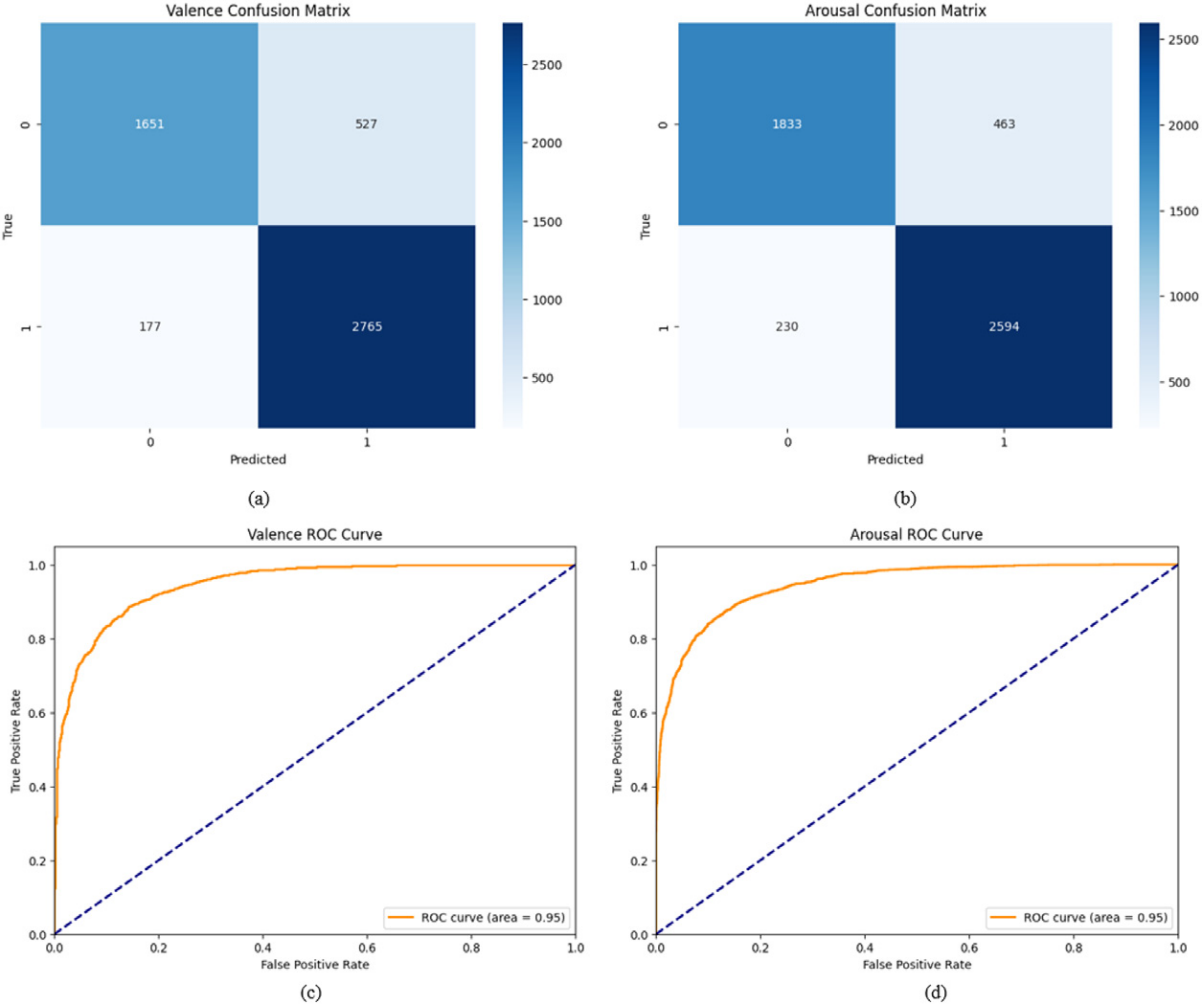
(a-b) Confusion matric and (c-d) ROC curve of Higuchi's fractal dimension using XGBoost algorithm.

The confusion matrices and ROC curve of differential entropy using XGBoost algorithm are shown in Fig. 9. Two distinct confusion matrices and ROC curves are created for the binary classification of valence and arousal task, respectively. Confusion matrices are essential for evaluating classification model performance by detailing TP, FP, TN and FN values. The ROC curve is a handy tool that shows the balance between the true positive rate and the false positive rate at different threshold settings. It provides a visual representation of how well a model performs in diagnosing across various classification thresholds. From Fig. 9 (c-d) we observe that, a valence state ROC curve area of 0.97 and an arousal state ROC curve area of 0.96 specifies that the model has excellent predictive performance in distinguishing between positive and negative classes for both valence and arousal, with values close to 1 suggesting high accuracy. Fig. 10 demonstrated the confusion matrices and ROC curve of Higuchi's fractal dimension using XGBoost algorithm for the binary classification of valence and arousal task respectively.

### Statistical analysis of differential entropy (DE) and Higuchi's fractal dimension (HFD) for emotion classification

Table 4 represents the statistical validation of Differential Entropy (DE) and Higuchi's Fractal Dimension (HFD) utilizing the XGBoost classifier. The table includes the mean and standard deviation (SD) values for Valence and Arousal, along with the associated p-values for each feature, confirming the discriminative power of DE and HFD for emotion classification. Paired *t*-tests on accuracy scores from a 5-fold cross-validation revealed that DE significantly outperforms HFD in both dimensions.

**Table 4:** Statistical analysis of DE and HFD using XGBoost.

Feature	Valence Mean	Valence SD	Arousal Mean	Arousal SD	p-value (valence)	p-value (Arousal)
DE	0.89554	0.02002	0.88420	0.01797	5.47562e-08	7.08739e-06
HFD	0.85820	0.03027	0.85729	0.02490	2.82540e-08	1.58306e-06

For Valence, DE achieved a mean accuracy of 0.89554 (SD = 0.02002), while HFD had a mean accuracy of 0.85820 (SD = 0.03027). The paired *t*-test yielded a highly significant p-value of 5.47562e-08 < 0.05 for DE, indicating its superior performance.

In Arousal, DE showed a mean accuracy of 0.88420 (SD = 0.01797), whereas HFD scored 0.85729 (SD = 0.02490). The p-value for DE was 7.08739e-06 < 0.05, further demonstrating its significant advantage over HFD, which had a p-value of 1.58306e-06. Since both p-values are far below the conventional alpha level of 0.05, the null hypotheses of equal mean accuracies are rejected for both Valence and Arousal.

These results confirm that DE provides a more reliable and superior feature for emotion classification tasks in both Valence and Arousal, highlighting its enhanced discriminative capability compared to HFD.

### Cross-Subject evaluation on SEED dataset

To address the limitations of within-subject validation and to better assess the generalization capabilities of our emotion recognition framework, we extended our experiments using the SEED dataset [35] and employed a cross-subject validation strategy. The SEED dataset contains EEG recordings from 15 subjects while watching film clips eliciting positive, neutral and negative emotions. For this study, we selected 10 participants and retained only positive and negative trials, discarding neutral ones to focus on binary emotion classification. A total of 32 EEG channels were selected to maintain consistency with the DEAP-based setup.

Each trial is approximately 4 min long and we segmented each into 20 non-overlapping temporal windows to capture the temporal evolution of emotional states. Each subject had 30 valid trials (15 positive, 15 negative), resulting in a total of:

• 30 trials × 20 segments = 600 segments per subject
• 600 × 10 = 6000 EEG segments in total

We extracted two time-domain features DE and HFD, from each EEG segment due to their effectiveness in modeling signal complexity and nonlinearity associated with emotional and cognitive dynamics. We adopted a Leave-One-Subject-Out (LOSO) cross-validation scheme, where the model is trained on EEG data from 9 subjects and evaluated on the remaining unseen subject. This process is repeated for all 10 subjects, and performance metrics are averaged to assess overall generalizability.

The results showing in Table 5, Table 6, clearly demonstrate the robustness of the proposed approach under subject-independent validation conditions. The XGBoost classifier consistently achieved the highest accuracy and F1 scores across both feature sets, with 86 % accuracy using HFD and 84 % using DE. These findings validate the effectiveness of using temporal EEG features such as DE and HFD for emotion recognition, particularly when combined with boosting techniques. The marginal drop in performance from the DEAP (within-subject) results to the SEED (cross-subject) results highlights the challenge of generalization across individuals, yet still indicates strong model reliability.

**Table 5:** Classification outcomes of differential entropy feature.

ML Model	Accuracy	Class 0 = Negative, 1 = Positive	Precision	Recall	F1 Score	Support
KNN	0.76	0	0.74	0.72	0.73	2967
		1	0.78	0.79	0.78	3033
SVM	0.73	0	0 0.70	0.71	0.70	2967
		1	0.75	0.74	0.74	3033
XGBoost	**0.84**	0	0.83	0.84	0.83	2967
		1	0.85	0.84	0.84	3033
Gradient Boosting	0.81	0	0.80	0.79	0.81	2967
		1	0.82	0.81	0.78	3033

**Table 6:** Classification outcomes of Higuchi’s fractal dimension feature.

ML Model	Accuracy	Class 0 = Negative, 1 = Positive	Precision	Recall	F1 Score	Support
KNN	0.78	0	0.76	0.77	0.76	2967
		1	0.80	0.79	0.80	3033
SVM	0.75	0	0 0.73	0.74	0.73	2967
		1	0.77	0.76	0.76	3033
XGBoost	**0.86**	0	0.85	0.86	0.85	2967
		1	0.87	0.84	0.83	3033
Gradient Boosting	0.83	0	0.82	0.81	0.82	2967
		1	0.84	0.83	0.83	3033

### Hyper-parameter tuning of the models

A model's resilience across many datasets and domains can be improved by fine-tuning its hyperparameters ensuring that it works well on novel and unexplored instances. In our study, we applied GridSearchCV technique to determine the best parameter values and this technique systematically investigates different hyperparameter combinations while evaluating their performance. Accuracy is the leading requirements in this investigation. Table 7 presents the best parameters found through grid search along with the corresponding best scores for the models.

**Table 7:** The Best Parameter and Best Score for The Model.

Models	Best parameter values	Best score	
		Valence	Arousal
KNN	n_neighbors = 5, metric = minkowski, Euclidean distance, *p* = 2, weights = uniform	78 %	77 %
SVM	Kernel = RBF, random_state = 42, *C* = 1, gamma = scale	63 %	60 %
XGBoost	Metric = mlogloss, label encoder = False, random_state = 42	89 %	88 %
Gradient Boosting	n_estimators = 100, random_state = 42	66 %	64 %

### Evaluation of the suggested approach considering leading-edge models

A comparison of our suggested method with current state-of-the-art techniques using the DEAP dataset for emotion recognition is shown in Table 8. This contrast emphasizes how crucial it is to use informative feature representations in addition to choosing suitable classifiers. Traditional studies have employed statistical, spectral or time-frequency features such as HOC, PSD and DWT, paired with classifiers like SVM and KNN. While some approaches achieved commendable accuracy, they often lacked the integration of features capable of capturing the nonlinear and complex nature of emotional brain responses.

**Table 8:** Performance comparison of proposed method with previous work utilizing DEAP dataset.

Research	Features	Classifier	Accuracy	
			Valence	Arousal
B. C. Alves et al. [33]	Spatial-frequency features	KNN, RF and SVM	85.05 %	85.46 %
N. Zhuang et al. [34]	Features extracted applying EMD	SVM	69.1 %	71.9 %
M. L. R. Menezes et al. [14]	Statistical Features, HOC and PSD	SVM	88.4 %	74 %
Z. Mohammadi et al. [18]	DWT	SVM and KNN	84.05 %	86.75 %
R. Nawaz et al. [19]	Statistical Features	SVM	Overall accuracy = 78.06 %	
Y. Fang et al. [20]	PSD and DE	MFDF	Overall accuracy = 71.05 %	
Md. Khateeb et al. [25]	Multi-domain Features	SVM	Overall accuracy = 65.92 %	
**Proposed work**	**DE and HFD**	**KNN, SVM, XGBoost and Gradient Boosting**	**89 %**	**88 %**

In contrast, our work leverages two powerful time-domain features Differential Entropy (DE) and Higuchi’s Fractal Dimension (HFD), which are specifically chosen for their ability to model complexity and dynamic changes in EEG signals. Furthermore, we combined these features with hyperparameter-tuned boosting algorithms, notably XGBoost, which significantly enhanced classification performance.

As demonstrated in Table 8, our method achieves a superior accuracy of 89 % for valence and 88 % for arousal, outperforming previous methods in both dimensions. This improvement reflects the synergy between advanced feature extraction techniques and ensemble learning models. Additionally, to ensure robustness and practical applicability, we validated our findings across two datasets (DEAP and SEED) and under both within-subject and cross-subject evaluation protocols, further demonstrating the generalizability of our approach.

## Conclusion

This study examined the application of diverse machine learning techniques to improve emotion classification from EEG signals, with particular emphasis on the DEAP dataset. We exploited two traditional classifiers, KNN and SVM in conjunction with two sophisticated boosting methods XGBoost and Gradient Boosting to enhance the model's efficacy. Due to the intrinsic temporal dependence of EEG signals, we employed a segmentation technique and subsequently extracted essential time-domain features: differential entropy and Higuchi's fractal dimension. These qualities were chosen for their significance in elucidating the fundamental patterns of emotional states.

We employed a 5-fold cross-validation procedure to assess the efficacy of the offered techniques. The findings illustrate the effectiveness of the XGBoost algorithm, with a remarkable classification accuracy of 89 % for the valence state and 88 % for the arousal condition. To further validate the robustness of our approach, we applied cross-subject evaluation using the SEED dataset, where the XGBoost classifier achieved 86 % accuracy using Higuchi’s Fractal Dimension and 84 % using Differential Entropy. These results highlight the effectiveness of combining hyperparameter-tuned, advanced machine learning models with feature extraction techniques for the categorization of emotions from EEG signals. The elevated accuracy attained in this study indicates that the proposed methodology is a potential avenue for emotion identification systems, with substantial practices in mental health monitoring, human-computer interaction and affective computing field. Future research may investigate the incorporation of supplementary signal processing methodologies and the refinement of model parameters to further improve classification efficacy. Furthermore, investigating more sophisticated deep learning techniques might lead to even greater advancements in the identification and interpretation of intricate emotional states from EEG signals.

## Limitations

Not applicable.

## Funding statement

This experiment was not supported in part by a grant.

## Supplementary material

The datasets used in the study are publicly accessible and can be found at the following links: • DEAP dataset [26] : https://www.eecs.qmul.ac.uk/mmv/datasets/deap/download.html • SEED dataset [35] : https://bcmi.sjtu.edu.cn/home/seed/downloads.html#seed-access-anchor

## CRediT authorship contribution statement

**Rabita Hasan:** Writing – original draft, Validation, Project administration, Methodology, Investigation, Formal analysis, Conceptualization. **Sheikh Md. Rabiul Islam:** Writing – review & editing, Validation, Project administration, Methodology, Investigation, Formal analysis, Conceptualization, Supervision.

## Declaration of competing interest

The authors declare that they have no known competing financial interests or personal relationships that could have appeared to influence the work reported in this paper.

## Data Availability

The data that has been used is confidential.
